# Effect of early physical therapy initiation and adherence on functional outcomes after arthroscopic rotator cuff repair: a prospective observational cohort study

**DOI:** 10.3389/fmed.2026.1743180

**Published:** 2026-04-01

**Authors:** Abdullah Raizah, Ravi Shankar Reddy, Mastour Saeed Alshahrani, Faisal M. Alyazedi, Batool Abdulelah Alkhamis, Saleh Kardm, Ghada Mohamed Koura, Devika Rani Sangadala, Debjani Mukherjee

**Affiliations:** 1 Department of Orthopaedic Surgery, College of Medicine, King Khalid University, Abha, Saudi Arabia; 2 Program of Physical Therapy, Department of Medical Rehabilitation Sciences, College of Applied Medical Sciences, King Khalid University, Abha, Saudi Arabia; 3 Physical Therapy Department, Prince Sultan Military College of Health Sciences, Dhahran, Saudi Arabia; 4 Department of Surgery, College of Medicine, Najran University, Najran, Saudi Arabia

**Keywords:** early mobilization, functional outcome, physical therapy, rehabilitation adherence, rotator cuff repair, shoulder function

## Abstract

**Introduction:**

Rotator cuff repair outcomes are influenced not only by surgical technique but also by postoperative rehabilitation. However, the optimal timing of physical therapy (PT) initiation and the role of adherence remain incompletely understood. This study aimed to evaluate the independent and combined effects of early PT initiation and adherence on functional recovery following arthroscopic rotator cuff repair.

**Methods:**

In this prospective observational cohort study, 160 patients undergoing arthroscopic rotator cuff repair were categorized into early (≤14 days) and delayed (>14 days) PT initiation groups. Functional outcomes were assessed at 3–6 months using validated measures, including the Disabilities of the Arm, Shoulder and Hand (DASH), Shoulder Pain and Disability Index (SPADI), Functional Independence Measure (FIM), and Visual Analog Scale (VAS). PT adherence was stratified by the number of sessions attended. Multivariate regression analysis was performed to identify independent predictors of functional outcomes.

**Results:**

Early PT initiation and higher adherence were independently associated with significantly improved functional outcomes, including lower DASH and SPADI scores, reduced pain, and greater range of motion (*p* ≤ 0.005). PT adherence emerged as the strongest predictor of DASH scores (β = −0.34, *p* < 0.001). Patients in the early PT group also demonstrated higher adherence levels and better functional independence. Subgroup analysis revealed that older age and larger tear size were associated with poorer outcomes, with a significant interaction effect.

**Discussion:**

Early initiation of PT combined with high adherence leads to superior functional recovery following arthroscopic rotator cuff repair. These findings highlight the importance of timely and structured rehabilitation protocols and suggest that patient-specific factors such as age and tear size should be considered when designing postoperative rehabilitation strategies.

## Introduction

Rotator cuff tears are among the most common causes of shoulder pain and dysfunction, particularly in middle-aged and older adults (1, 2). Arthroscopic rotator cuff repair has become the standard surgical approach for treating symptomatic, full-thickness tears, offering improved visualization, reduced morbidity, and favorable clinical outcomes (3). However, surgical repair alone does not guarantee optimal recovery (4). Postoperative rehabilitation, especially physical therapy (PT), plays a pivotal role in restoring shoulder function, reducing pain, and facilitating return to daily activities and work (5). The timing and consistency of PT delivery are widely recognized as modifiable factors that may influence recovery trajectories (5). Nevertheless, clinical practice remains variable regarding when to initiate rehabilitation and how best to ensure patient adherence to prescribed regimens (6).

Several studies have explored the impact of early versus delayed PT initiation on postoperative outcomes following rotator cuff repair (6, 7). Some evidence suggests that early rehabilitation facilitates improved short-term range of motion and pain relief without compromising tendon healing integrity (8). Hu et al. (8) reported that early passive motion enhanced functional outcomes without increasing re-tear rates. Similarly, Kjær et al. (9) demonstrated a superior range of motion and pain control in patients who commenced therapy earlier. In parallel, adherence to rehabilitation protocols has emerged as a critical determinant of long-term recovery. Kjær et al. (9) and Ziedas et al. (10) both identified a positive association between higher adherence and improved patient-reported outcomes. However, much of the existing research evaluates timing or adherence in isolation, and there remains limited integration of these variables in a comprehensive analysis (6, 7). Moreover, few studies have examined how factors such as age, tear size, or comorbidities may modify the impact of PT timing and adherence on functional outcomes (4, 11).

Given the complex, multifactorial nature of recovery following rotator cuff repair, a deeper understanding of how early initiation and sustained adherence to PT interact with patient-specific characteristics is clinically essential (12). There is a need for data-driven guidance to inform individualized rehabilitation protocols. In particular, the interaction between early rehabilitation and adherence remains underexplored in the context of diverse clinical profiles (12, 13). While studies have shown that early PT and adherence are beneficial, the extent to which these factors collectively influence pain, function, and independence—especially across varying age groups and tear severities—warrants further investigation (6, 7). Additionally, most prior studies have not quantified the effect sizes of these variables using multivariate models that account for common confounders such as diabetes, smoking status, and surgical technique. Clarifying these relationships is essential for optimizing postoperative protocols and improving patient-centered outcomes.

The objective of this study was to evaluate the independent and combined effects of early PT initiation and adherence on functional outcomes at 3–6 months following arthroscopic rotator cuff repair. A secondary aim was to identify clinical and demographic predictors of superior recovery, with a focus on age and tear size as potential effect modifiers. It was hypothesized that earlier PT initiation and higher adherence would be associated with significantly better functional outcomes, as measured by validated instruments such as the Disabilities of the Arm, Shoulder and Hand (DASH) score, Shoulder Pain and Disability Index (SPADI), and Functional Independence Measure (FIM), and that older age and larger tear size would negatively influence these outcomes.

## Methods

### Study design, ethics, and settings

This prospective observational cohort study was conducted between April 2024 and February 2025. Ethical approval for the study was granted by the Institutional Review Board of King Khalid University (Approval Number: REC-349-2024). All participants provided written informed consent prior to enrollment. The study was conducted in accordance with the ethical standards of the Declaration of Helsinki.

### Participants

Participants were recruited exclusively from the Orthopedic Outpatient Clinic at DMRS, KK University (Abha). All assessments, surgical procedures, and postoperative rehabilitation were conducted at this single site, following standardized protocols established within the institution. Eligible individuals were referred for postoperative rehabilitation following arthroscopic rotator cuff repair. Diagnosis and surgical confirmation of full-thickness rotator cuff tears were based on clinical evaluation, corroborated by magnetic resonance imaging (MRI) (14). All surgeries were performed using standard arthroscopic techniques by board-certified orthopedic surgeons (14). Inclusion criteria included adults aged 40–70 years who underwent isolated arthroscopic repair for a single or multi-tendon full-thickness rotator cuff tear, with the ability to participate in structured physical therapy within 6 weeks after surgery. Participants needed to have sufficient cognitive capacity to follow rehabilitation instructions and complete outcome measures independently. Exclusion criteria included individuals with prior shoulder surgeries, revision repairs, concomitant shoulder pathologies such as adhesive capsulitis or labral tears, bilateral shoulder involvement, neurological deficits affecting upper limb function, or systemic conditions that interfered with rehabilitation (such as advanced cardiopulmonary disease or uncontrolled diabetes mellitus). Patients were screened through clinical records and postoperative evaluations to confirm eligibility, and baseline assessments were conducted prior to the initiation of physical therapy. These included demographic profiling, pre-injury activity levels using the UCLA Activity Score, and baseline functional disability via the DASH questionnaire. Only patients who agreed to participate and completed at least one follow-up assessment within 3–6 months post-surgery were included in the final analysis.

### Disabilities of the arm, shoulder, and hand score

The DASH questionnaire was utilized as the primary measure of upper extremity functional impairment in this study (15). Developed by the Institute for Work and Health and endorsed by the American Academy of Orthopedic Surgeons (AAOS), the DASH is a validated, patient-reported outcome measure specifically designed to assess physical function and symptoms in individuals with upper limb musculoskeletal disorders (15). It comprises 30 items rated on a 5-point Likert scale (1 = no difficulty or symptoms; 5 = extreme difficulty or symptoms), capturing the severity of disability during daily activities, pain, tingling, sleep disturbances, and the impact on social functioning (15). At least 27 items must be completed for a valid score, which is calculated using the standardized formula: [(∑(Xi − 1))/(*n* − 1)] × 25, yielding a total score from 0 (no disability) to 100 (most severe disability). The DASH was administered once, between 3 and 6 months postoperatively, with data collected in person by a blinded research assistant. The Arabic version of the DASH, previously culturally adapted and psychometrically validated for post-rotator cuff repair populations, was used; this version has demonstrated excellent reliability (Cronbach’s α > 0.90; ICC > 0.85) (16). Participants completed the questionnaire independently under supervision, and any incomplete responses were handled in accordance with the official DASH user manual.

### Shoulder pain and disability index

The SPADI was utilized in this study as a validated, patient-reported outcome measure to evaluate shoulder-specific pain and functional disability following arthroscopic rotator cuff repair (17). The SPADI consists of 13 items divided into two subscales: pain (5 items) and disability (8 items). Each item is rated on a 0–10 numerical scale, where 0 represents “no pain” or “no difficulty” and 10 indicates “worst pain imaginable” or “so difficult it requires help.” Total SPADI scores are calculated by averaging the scores of all completed items and expressing the result as a percentage, yielding a score from 0 (no pain or disability) to 100 (maximum pain and disability) (17). A minimum of at least 12 of the 13 items must be completed to yield a valid score, with the pain and disability subscale scores also calculated separately when applicable. In this study, the Arabic version of the SPADI (18), which has been cross-culturally adapted and psychometrically validated in shoulder rehabilitation populations, was employed. It has demonstrated strong internal consistency (Cronbach’s α > 0.89) and excellent test–retest reliability (ICC > 0.90) in previous post-operative shoulder cohorts (18). Participants completed the SPADI at 3–6 months postoperatively, supervised by a trained and blinded research assistant to ensure accuracy and completeness. Missing data were managed according to the published scoring guidelines.

### Functional independence measure (FIM—upper limb subscale)

The FIM—Upper Limb Subscale was employed to assess patients’ level of functional independence in activities of daily living (ADLs) involving the affected upper extremity (19). The FIM instrument is a widely validated functional assessment tool composed of 18 items across motor and cognitive domains; however, this study focused specifically on the self-care and upper limb motor domains relevant to shoulder function (19). Each item is scored on a 7-point ordinal scale, ranging from 1 (total assistance required) to 7 (complete independence), with higher scores indicating greater independence. The maximum attainable score for upper limb-related tasks was 21 points (19). Assessments were performed at 3–6 months postoperatively by a blinded clinician trained in standardized FIM administration procedures (19). The measure’s ability to capture clinically meaningful changes in patient function over time made it an appropriate outcome for this analysis.

### Visual analog scale for pain

Pain intensity was evaluated using the Visual Analog Scale (VAS), a validated, unidimensional tool designed to assess a patient’s current level of shoulder pain (20). Participants were asked to rate their average pain over the previous 24 h by marking a point along a 10-centimeter horizontal line, anchored by “no pain” (0) on the left and “worst imaginable pain” (10) on the right. The distance in centimeters from the left end to the patient’s mark was measured and recorded as the VAS pain score. This tool has demonstrated reliability (ICC > 0.80) in post-operative orthopedic populations, including those recovering from rotator cuff repair (21). The VAS was administered at the 3–6 month postoperative follow-up by a blinded assistant under standardized conditions to ensure consistency.

### Patient global impression of change

The Patient Global Impression of Change (PGIC) scale was used as a subjective, patient-centered measure of perceived overall recovery following surgery (22). The PGIC is a 7-point Likert scale that captures the patient’s self-assessment of improvement since the start of treatment, ranging from 1 (“very much improved”) to 7 (“very much worse”) (22). For analytical purposes in this study, responses were categorized into three groups: Improved (scores 1–2), Unchanged (scores 3–5), and Worsened (scores 6–7). This approach is consistent with previous research in musculoskeletal rehabilitation and allows for a meaningful comparison of subjective recovery between groups (23). The PGIC was administered during the 3–6 month follow-up in a supervised setting to ensure comprehension and accuracy. The scale has been validated for use in orthopedic populations and is recommended by international guidelines as a global anchor for interpreting the clinical relevance of outcome measures.

### Return to work and use of assistive devices

Return to work status and the use of assistive devices were recorded as dichotomous variables (Yes/No) based on structured interviews conducted at the 3–6 month follow-up. Patients were asked whether they had returned to their pre-injury occupational duties and whether they required any external assistive devices (slings, canes) for daily upper limb function. These variables were included as indicators of real-world functional recovery and independence. Although not scored using standardized scales, they provided clinically relevant insight into rehabilitation outcomes and were analyzed descriptively and inferentially across study groups. Return to work is a commonly reported endpoint in orthopedic rehabilitation studies, often reflecting a composite of physical recovery, psychosocial readiness, and work demands.

### Independent variables/predictors

The two main predictors investigated were timing of physical therapy initiation and level of physical therapy adherence. Time to PT initiation was recorded as the number of days from surgery to the first supervised PT session. Participants were then categorized into two groups: early PT initiation (≤14 days post-surgery) and delayed PT initiation (>14 days post-surgery), based on prior literature and clinical relevance to early rehabilitation windows (7, 8). Group classification was based on the observed timing of rehabilitation initiation in routine clinical practice and was not determined by random allocation or surgeon-directed assignment. The timing of the first PT session was primarily influenced by scheduling logistics, patient availability, transportation considerations, and clearance at the initial postoperative follow-up visit. No insurance-based or protocol-mandated criteria were used to assign patients to early or delayed rehabilitation. This approach reflects real-world variability in rehabilitation access within a standardized institutional care pathway. This 14-day threshold was selected based on prior randomized controlled trials and meta-analyses that identified the first two postoperative weeks as a pivotal period for initiating passive and assisted mobilization without increasing the risk of re-tear (7, 8). Although a two-day individual-level cutoff may seem narrow, the actual mean difference between groups was over 11 days, which reflects a clinically meaningful divergence in rehabilitation exposure. The chosen classification is consistent with previously validated timelines and enhances comparability with existing evidence. Broader categories, such as 0–3 weeks or 0–6 weeks, may dilute early-phase effects that are hypothesized to influence neuromuscular recovery during the acute postoperative period. Adherence was measured based on the total number of attended sessions within the first 3 months and categorized into three levels: high adherence (≥15 sessions), moderate adherence (10–14 sessions), and low adherence (<10 sessions). These thresholds were selected based on previous rehabilitation studies demonstrating dose-dependent outcomes. Additional predictors examined via multivariate regression included age, sex, BMI, tear size (small/medium/large), smoking history, comorbid diabetes, and time since surgery. Time since surgery refers to the interval between the date of arthroscopic rotator cuff repair and the final follow-up visit (at which outcome measures were assessed), representing the postoperative follow-up duration rather than the time to physical therapy initiation.

### Physiotherapy program components and home exercise adherence

The structured physiotherapy program followed a standardized three-phase progression over 12 weeks. Phase I (Weeks 1–4) focused on protected passive and active-assisted range of motion (ROM), including pendulum exercises, passive external rotation, and scapular mobilization, combined with cryotherapy and patient education. During this phase, all movements were strictly limited to pain-free, therapist-assisted or passive exercises performed within surgeon-defined ROM parameters. Active shoulder elevation against gravity, resisted strengthening, and loaded movements were not permitted. The abduction sling, provided immediately postoperatively, was worn continuously for 4 weeks and removed only during supervised therapy sessions and personal hygiene. All exercises were performed in supported positions to minimize tensile stress on the repair site, and patients were instructed to avoid active shoulder motion and lifting activities. For patients with large tears, ROM limits—particularly in forward elevation and external rotation—were more conservative, and progression to active motion was deferred until formal surgical clearance at the 4–6-week follow-up. Strengthening for this subgroup was initiated only after confirmation of satisfactory early healing and clinical stability.

Phase II (Weeks 5–8) involved progression to active ROM, introduction of isometric strengthening of the rotator cuff and periscapular musculature, and postural training. Phase III (Weeks 9–12) emphasized progressive isotonic strengthening using resistance bands and functional task simulation, such as reaching and lifting activities. Advancement between phases was contingent upon pain control, absence of compensatory movement patterns, and surgeon approval, thereby prioritizing biological healing and repair protection throughout rehabilitation. Manual therapy techniques were selectively incorporated across phases to address soft-tissue restrictions and optimize scapulothoracic mechanics.

All participants followed this identical three-phase protocol; however, the timing of entry into Phase I differed between groups. Patients in the early PT group-initiated Phase I within 14 days postoperatively, whereas those in the delayed PT group began the same protocol after this period. Thus, group allocation reflected differences in rehabilitation onset rather than treatment content, allowing evaluation of the impact of timing on functional recovery.

Routine postoperative care included standardized analgesic management with NSAIDs and cryotherapy, suture inspection at 7–10 days, and surgical follow-up visits at 2 and 6 weeks to assess wound healing and authorize progression of rehabilitation phases. A structured home exercise program was prescribed concurrently with supervised therapy and adjusted according to each recovery phase. Exercises included pendulum movements, wand-assisted flexion and external rotation, scapular retraction drills, wall crawls, and resisted abduction using elastic bands as appropriate to phase progression. Patients were instructed to perform home exercises twice daily, with weekly monitoring and progression by physiotherapists. Adherence was assessed through self-reported exercise logs documenting frequency and completion of prescribed sessions; compliance was calculated as the percentage of prescribed exercises performed and reviewed regularly to guide individualized program modifications.

### Covariates/confounders

To control for potential confounding, variables including pre-injury activity level (UCLA Activity Score), occupation type (manual/sedentary), dominant arm involvement, and surgical technique (single vs. double-row repair) were recorded. Pre-injury activity was assessed using the UCLA score (range: 1–10), with higher values indicating more active lifestyles. Tear size was categorized intraoperatively based on arthroscopic visualization and standard classification guidelines.

### Data collection instruments and procedures

All measurements were conducted by trained physiotherapists blinded to group assignment to minimize bias. Standardized protocols were followed for goniometry, questionnaire administration, and adherence scoring. Patient-reported outcomes were collected using validated Arabic-language versions of DASH and SPADI where applicable. Therapist-rated functional progress was scored using a 10-point Likert scale based on observed clinical milestones. At the same time, patient-reported home exercise compliance was self-reported as a percentage of prescribed sessions completed. The scale ranged from 1 (minimal progress) to 10 (optimal progress), based on observed task performance and progression milestones. Ratings were performed at each PT visit by the same therapist trained in standardized scoring procedures to ensure inter-rater consistency. Data were recorded in electronic case report forms and cross-verified for accuracy before analysis.

### Sample size calculation

The sample size was calculated using *G*Power 3.1.9.7* for an ANCOVA comparing DASH scores between early and delayed physical therapy initiation groups after arthroscopic rotator cuff repair (24). The test family selected was the F test, with the statistical test ANCOVA: Fixed effects, main effects, and interactions. The model included two groups (early vs. delayed PT) and two covariates (baseline DASH score and pre-injury activity level), yielding a total of 3 predictors. The effect size was set to *f* = 0.25 (medium), with α = 0.05 and power = 0.80. The number of covariates was set to 2, and the numerator degrees of freedom was set to 1, as specified by the model structure. Assuming a medium effect size (*f* = 0.25), alpha = 0.05, power = 0.80, and an equal allocation ratio (1:1), a minimum of 128 participants (64 per group) was required. To accommodate a 20% potential data loss, the final target sample size was set at 160 participants, ensuring adequate power for the primary and secondary analyses.

### Data analysis

All statistical analyses were conducted using IBM SPSS Statistics software, version 24.0 (IBM Corp., Armonk, NY, United States). Continuous variables were tested for normality using the Shapiro–Wilk test and found to be normally distributed; hence, parametric tests were employed. Descriptive statistics were reported as means and standard deviations for continuous variables and frequencies with percentages for categorical variables. Independent samples *t*-tests were used to compare continuous outcomes (DASH, SPADI, FIM scores, range of motion, and pain scores) between the early and delayed PT groups. Chi-square tests were applied to categorical variables, including adherence levels, PGIC ratings, return to work, and assistive device use. One-way ANOVA followed by Bonferroni *post hoc* tests was used to compare functional outcomes across PT adherence categories (high, moderate, and low). Multiple linear regression was performed to identify independent predictors of DASH scores at 3–6 months, including age, sex, BMI, time to PT initiation, adherence, comorbidities, and tear size, with interaction terms tested where relevant. Given that only one postoperative follow-up assessment was conducted per participant (at 3–6 months), there were no repeated time points available to model within-subject change. Consequently, a linear mixed-effects model could not be used to examine a group × time interaction, as no longitudinal structure existed in the dataset. While mixed-effects models are highly flexible and can accommodate complex random effects structures even in 2 × 2 designs, their primary advantage lies in modeling variability across repeated observations, which was not possible in this cross-sectional design. Thus, multiple linear regression was employed to account for covariates and estimate the independent effects of PT timing and adherence on outcomes. However, we acknowledge that a linear mixed-effects model incorporating fixed and random effects could more comprehensively account for variability across patients and covariates in future studies that include multiple time points or hierarchical data. Subgroup analyses were conducted using two-way ANOVA to examine the interaction effects of age and tear size on functional outcomes. Prior to regression, outlier diagnostics were conducted using standardized residuals, leverage statistics, and Cook’s distance; no data points exceeded standard exclusion criteria (residual > ± 3.0, Cook’s D > 1.0). Assumptions of linearity, independence, homoscedasticity, and normality of residuals were assessed through scatterplots of residuals, normal probability plots, and Shapiro–Wilk tests. Multicollinearity was checked using variance inflation factors (VIF), with all predictors showing VIF < 2.0. Given that the study design involved a single postoperative follow-up without repeated measures, mixed-effects modeling was not applicable. Future studies incorporating longitudinal measurements may benefit from hierarchical or mixed-model approaches to better account for within-subject variability over time. All statistical tests were two-tailed, and a *p*-value < 0.05 was considered statistically significant.

## Results

The demographic and clinical characteristics of participants were broadly balanced between the early and delayed PT groups, with no significant differences observed in age, sex, BMI, occupation type, comorbidities, or surgical technique (Table 1). However, patients in the early PT group demonstrated significantly higher pre-injury activity levels (UCLA score: 6.84 ± 1.43 vs. 6.42 ± 1.65, *p* = 0.041), a greater number of completed PT sessions (14.25 ± 3.64 vs. 11.84 ± 3.89, *p* = 0.004), and notably earlier PT initiation (8.34 ± 2.31 vs. 19.42 ± 3.56 days, *p* < 0.001). Additionally, baseline functional disability was significantly lower in the early PT group as indicated by DASH scores (46.73 ± 10.29 vs. 52.19 ± 11.02, *p* < 0.001), suggesting that earlier rehabilitation may be associated with better initial shoulder function (Table 1).

**Table 1:** Demographic and clinical characteristics of the study population.

Variable	Early PT (*n* = 80)	Delayed PT (*n* = 80)	*p*-value
Age (years)	58.42 ± 8.76	59.87 ± 9.01	0.356
Sex (male/female)	46/34	44/36	0.713
BMI (kg/m^2^)	27.89 ± 3.15	28.12 ± 3.47	0.482
Occupation type (manual/sedentary)	32/48	28/52	0.592
Pre-injury activity level (UCLA score)	6.84 ± 1.43	6.42 ± 1.65	0.041
Time from injury to surgery (weeks)	5.73 ± 2.11	6.38 ± 2.45	0.083
Surgical technique (single/double row)	37/43	34/46	0.651
Anchor type (metallic/bioabsorbable)	45/35	42/38	0.411
Dominant arm affected (yes)	51 (63.75%)	47 (58.75%)	0.527
Tear size (small/medium/large)	21/43 / 16	19/45 / 16	0.983
Diabetes mellitus (yes)	18 (22.50%)	21 (26.25%)	0.549
Hypertension (yes)	33 (41.25%)	37 (46.25%)	0.412
Smoking history (yes)	21 (26.25%)	25 (31.25%)	0.321
Time since surgery (months)	4.78 ± 0.92	4.89 ± 1.05	0.614
Time to PT initiation (days)	8.34 ± 2.31	19.42 ± 3.56	<0.001
PT sessions completed (n)	14.25 ± 3.64	11.84 ± 3.89	0.004
Baseline DASH score	46.73 ± 10.29	52.19 ± 11.02	<0.001

PT, Physical Therapy; BMI, Body Mass Index; DASH, Disabilities of the Arm, Shoulder, and Hand; UCLA, University of California at Los Angeles Activity Score; ADLs, Activities of Daily Living; SD, Standard Deviation.

Significant differences in PT initiation and adherence patterns were observed between groups, with earlier PT recipients starting rehabilitation nearly 11 days sooner and attending more sessions overall (*p* < 0.001 and *p* = 0.004, respectively) (Table 2). The early PT group also demonstrated greater session frequency, longer duration per session, and fewer missed appointments, reflecting superior adherence profiles (*p* ≤ 0.026). A significantly higher proportion achieved high adherence (≥15 sessions), and engagement in home exercise programs was more prevalent (*p* = 0.017 and *p* = 0.034, respectively). Moreover, early PT participants reported significantly higher patient-reported adherence and received better therapist-rated compliance scores (*p* = 0.002 and *p* = 0.005). No significant differences were noted in PT delivery mode or use of pain medications between groups (Table 2).

**Table 2:** Physical therapy initiation and adherence patterns.

Variable	Early PT (*n* = 80)	Delayed PT (*n* = 80)	*p*-value
Time to first PT session (days)	8.34 ± 2.31	19.42 ± 3.56	<0.001
PT sessions attended (mean ± SD)	14.25 ± 3.64	11.84 ± 3.89	0.004
PT sessions attended (median [IQR])	14 [12–16]	12 [9–14]	–
PT frequency per week	2.83 ± 0.62	2.39 ± 0.58	0.001
Session duration (minutes)	42.13 ± 5.77	39.82 ± 6.03	0.026
No. of missed sessions	1.23 ± 0.87	1.92 ± 1.05	0.009
Adherence level (high ≥15/moderate 10–14/low <10)	45/28 / 7	31/29 / 20	0.017
Mode of PT delivery (in-person/hybrid/telehealth)	65/10 / 5	58/12 / 10	0.381
Use of pain medications (NSAIDs/opioids/none)	51/12 / 17	43/18 / 19	0.241
Home exercise program (yes)	62 (77.50%)	49 (61.25%)	0.034
Patient-reported adherence (%)	84.62 ± 11.34	75.46 ± 13.92	0.002
Therapist-rated compliance (score: 1–10)	8.71 ± 1.12	7.83 ± 1.38	0.005

PT, Physical Therapy; NSAIDs, Non-Steroidal Anti-Inflammatory Drugs; IQR, Interquartile Range; SD, Standard Deviation.

**Figure 1:**
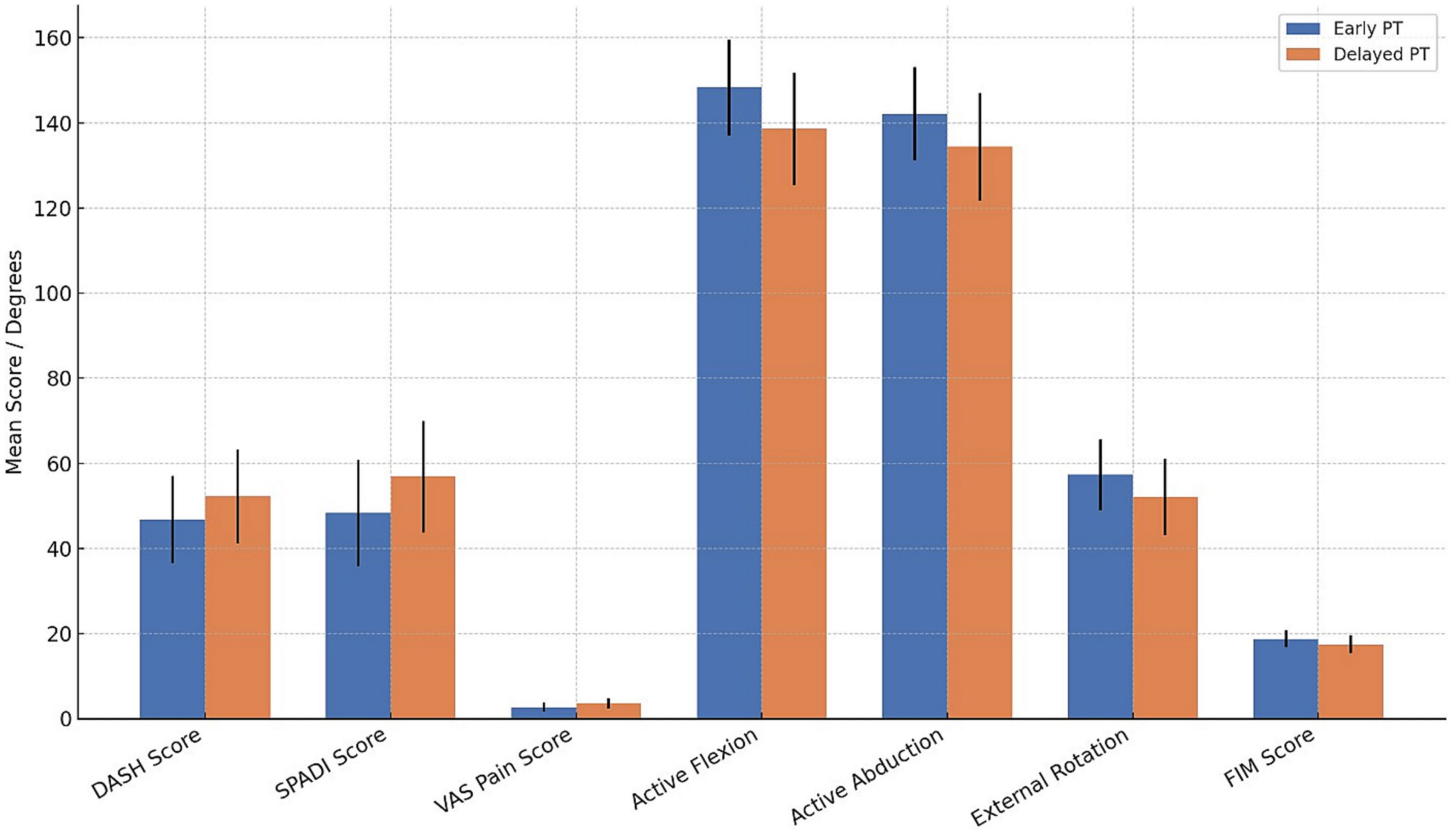
Comparison of functional outcomes between early and delayed physical therapy groups at 3–6 months post-arthroscopic rotator cuff repair.

At 3–6 months post-arthroscopic rotator cuff repair, patients who initiated physical therapy earlier demonstrated significantly better functional outcomes across nearly all measured domains, including lower DASH and SPADI scores (mean differences exceeding their respective minimal clinically important differences), reduced pain (VAS), and superior shoulder range of motion in flexion, abduction, and external rotation (*p* ≤ 0.005 for all) (Figure 1). Functional independence, as measured by FIM scores, was also significantly higher in the early PT group (*p* = 0.008), and a greater proportion of patients reported subjective improvement via PGIC ratings (*p* = 0.041). Differences in return-to-work rates and use of assistive devices were not statistically significant, though trends favored early rehabilitation.

**Figure 2:**
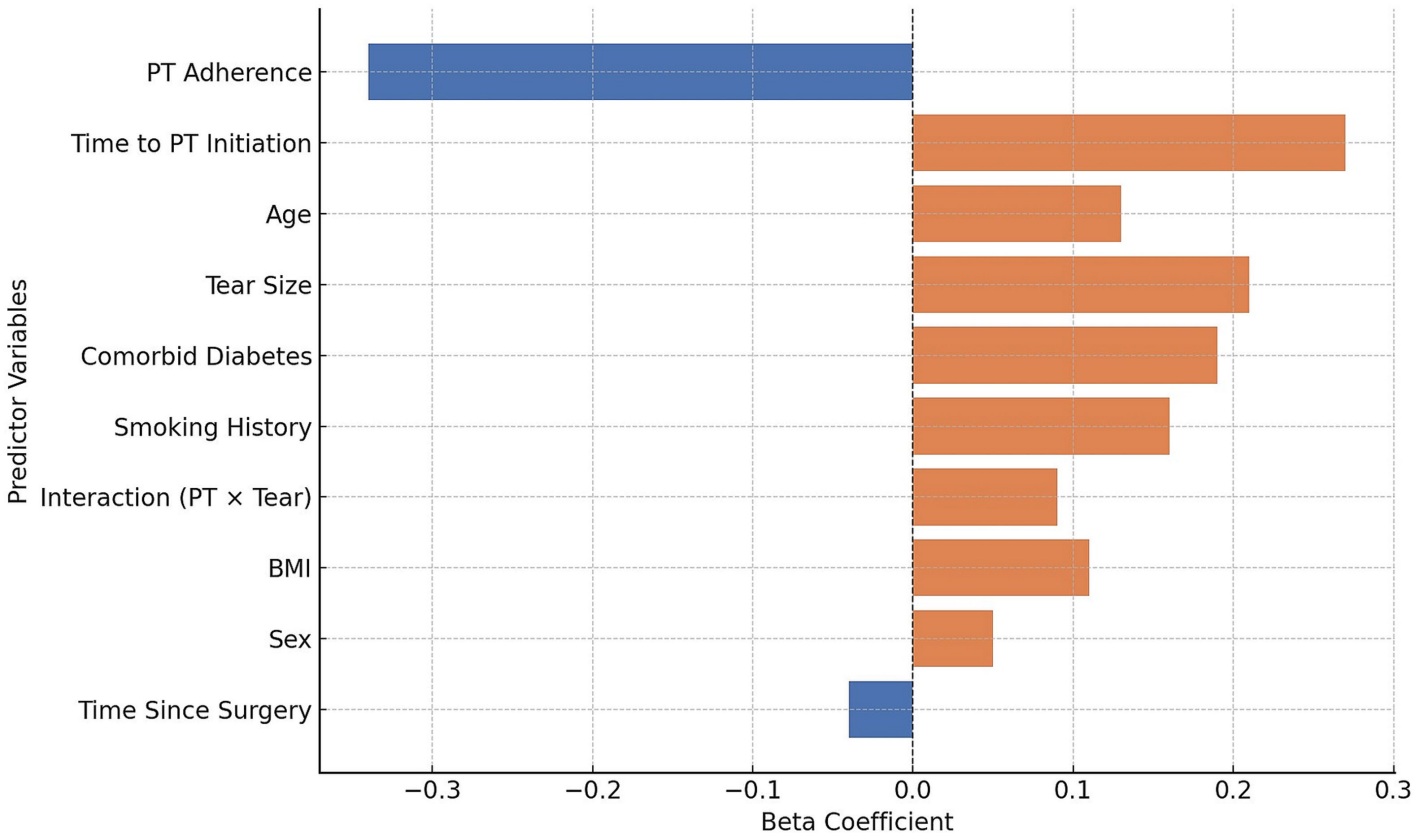
Standardized beta coefficients from multiple linear regression predicting DASH scores at 3–6 months post-arthroscopic rotator cuff repair.

Multiple linear regression analysis identified several significant predictors of better functional outcomes, as indicated by lower DASH scores, at 3–6 months post-surgery (Table 3; Figure 2). Earlier initiation of physical therapy and greater adherence were independently associated with improved outcomes, with PT adherence showing the largest effect size (β = −0.34, *p* < 0.001). Increasing age, larger tear size, presence of diabetes, and a history of smoking were all associated with worse functional scores (*p* < 0.05). The interaction between time to PT and tear size was also significant (β = 0.09, *p* = 0.033), suggesting that delayed therapy may be particularly detrimental in patients with more extensive pathology. The model explained 41% of the variance in DASH scores (adjusted *R*^2^ = 0.37), with acceptable multicollinearity and normally distributed residuals.

**Table 3:** Multiple linear regression analysis for predictors of better functional outcome (lower DASH score) at 3–6 months post-surgery.

Variable	Beta coefficient	Standard error	95% CI (lower–upper)	*p*-value
Time to PT initiation (days)	0.27	0.08	0.11–0.43	0.001
PT adherence (sessions completed)	−0.34	0.09	−0.52 to −0.16	<0.001
Age (years)	0.13	0.06	0.01–0.25	0.032
Sex (male = 0, female = 1)	0.05	0.05	−0.04 to 0.14	0.256
BMI (kg/m^2^)	0.11	0.07	−0.03 to 0.25	0.121
Tear size (small = 0, medium = 1, large = 2)	0.21	0.08	0.05–0.37	0.011
Comorbid diabetes (no = 0, yes = 1)	0.19	0.09	0.01–0.37	0.041
Smoking history (no = 0, yes = 1)	0.16	0.07	0.02–0.30	0.027
Time since surgery (months)	−0.04	0.06	−0.16 to 0.08	0.489
Interaction: time to PT × tear size	0.09	0.04	0.01–0.17	0.033

PT, Physical Therapy; DASH, Disabilities of the Arm, Shoulder and Hand; BMI, Body Mass Index; CI, Confidence Interval; SD, Standard Deviation.

Multicollinearity was assessed using variance inflation factors (VIF < 2.0 for all variables); residuals showed normal distribution and homoscedasticity on visual inspection.

Model *R*^2^ = 0.41; Adjusted *R*^2^ = 0.37.

**Figure 3:**
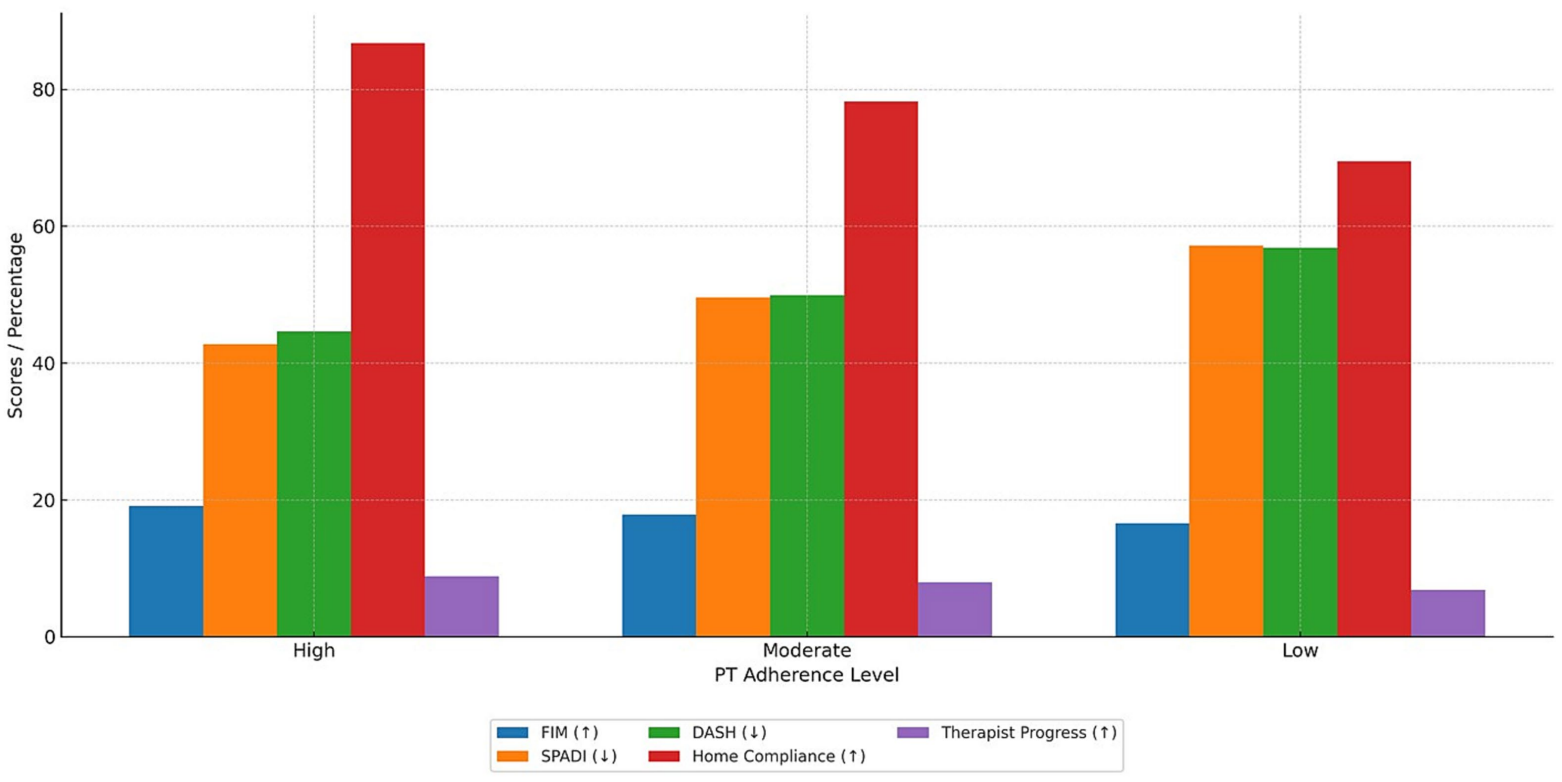
Physical therapy adherence levels and associated functional outcomes at 3–6 months post-arthroscopic rotator cuff repair.

Higher PT adherence was consistently associated with superior functional outcomes and greater independence across all measured domains at 3–6 months post-surgery (Figure 3). Patients in the high adherence group achieved significantly higher FIM scores and reported lower disability on SPADI and DASH scales compared to those with moderate or low adherence (*p* < 0.001 for all). Time to return to independent ADLs was shortest in the high adherence group (median 4 weeks), and both patient-reported home compliance and therapist-rated functional progress scores showed a clear stepwise improvement with increasing adherence levels. The effect size for key outcomes was substantial (Cohen’s d = 1.12 for high vs. low adherence), underscoring the clinical importance of sustained engagement with rehabilitation protocols.

**Figure 4:**
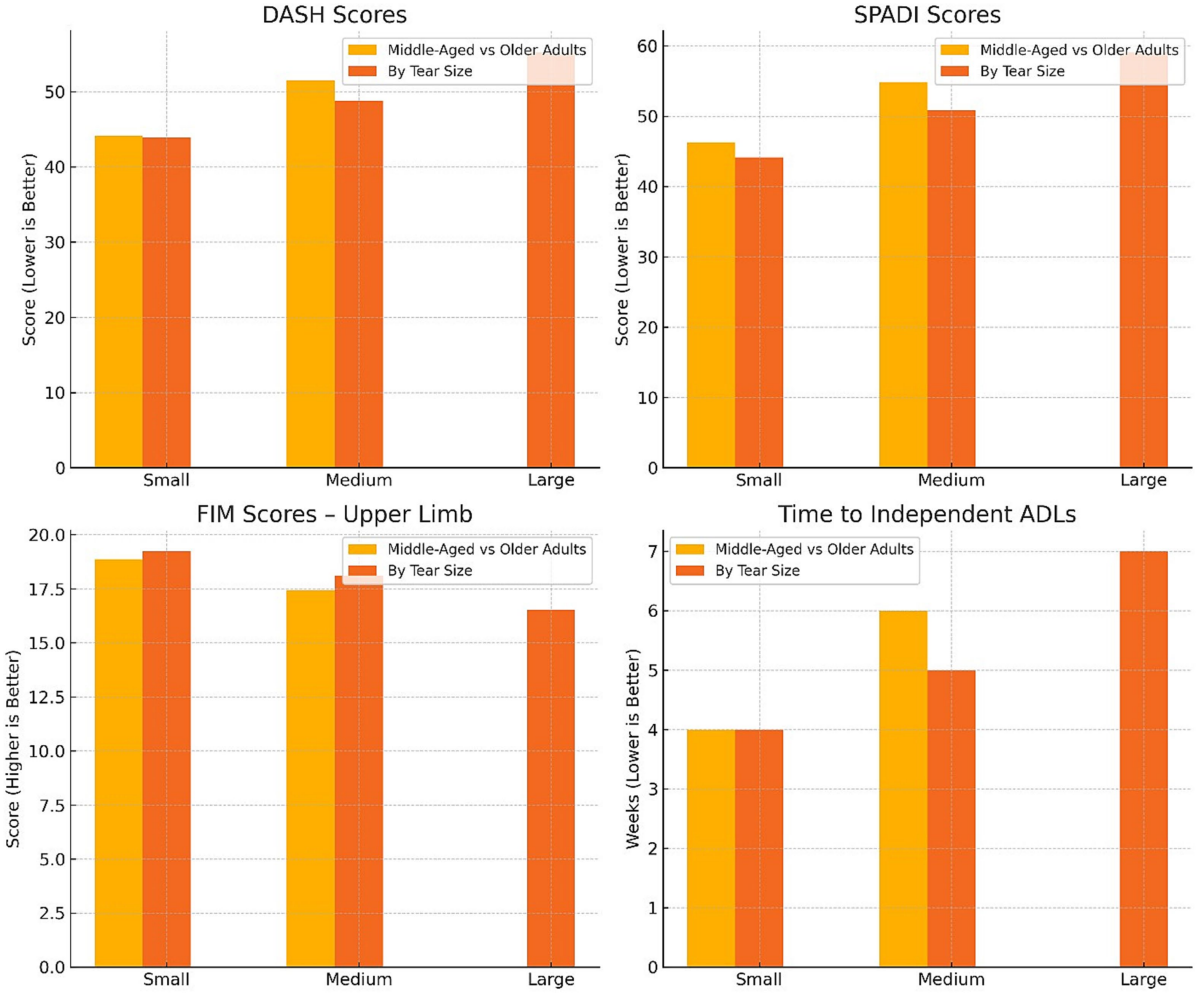
Subgroup comparison of functional outcomes by age group and tear size at 3–6 months post-arthroscopic rotator cuff repair.

Subgroup analysis revealed that both older age and larger tear size were independently associated with significantly poorer functional outcomes, as indicated by higher DASH and SPADI scores, lower FIM scores, and longer time to regain independence in ADLs (Table 4; Figure 4). Middle-aged patients (40–54 years) consistently outperformed older adults (55–70 years) across all functional measures (*p* < 0.001 for DASH, SPADI, and FIM), while patients with small tears had more favorable outcomes than those with medium or large tears (*p* < 0.001 for all comparisons). A statistically significant interaction effect was identified between age and tear size, suggesting that older patients with larger tears are disproportionately affected in terms of functional recovery (DASH: *p* = 0.039; SPADI: *p* = 0.045; FIM: *p* = 0.028). These findings underscore the compounded impact of age and injury severity on postoperative rehabilitation trajectories.

**Table 4:** Subgroup analysis of functional outcomes by age group and rotator cuff tear size.

Variable	Middle-aged (40–54 years, *n* = 70)	Older adults (55–70 years, *n* = 94)	*p*-value (age group)	Small tear (*n* = 40)	Medium tear (*n* = 88)	Large tear (*n* = 36)	*p*-value (tear size)	Interaction (age × tear size)
DASH score (mean ± SD, 95% CI)	44.12 ± 9.35 (42.01–46.23)	51.47 ± 10.63 (49.21–53.73)	<0.001	43.92 ± 8.47 (41.35–46.49)	48.73 ± 9.82 (46.80–50.66)	55.28 ± 11.06 (52.09–58.47)	<0.001	*p* = 0.039
SPADI score (mean ± SD, 95% CI)	46.28 ± 10.84 (43.73–48.83)	54.79 ± 12.17 (52.08–57.50)	<0.001	44.13 ± 9.96 (41.35–46.91)	50.86 ± 11.32 (48.30–53.42)	59.14 ± 12.88 (55.53–62.75)	<0.001	*p* = 0.045
FIM score – upper limb (mean ± SD, 95% CI)	18.86 ± 1.58 (18.48–19.24)	17.43 ± 1.91 (17.01–17.85)	<0.001	19.24 ± 1.37 (18.85–19.63)	18.12 ± 1.64 (17.77–18.47)	16.53 ± 2.03 (15.96–17.10)	<0.001	*p* = 0.028
Time to independent ADLs (weeks, median [IQR])	4 [3–6]	6 [4–8]	0.002	4 [3–5]	5 [4–6]	7 [6–9]	0.001	–

DASH, Disabilities of the Arm, Shoulder, and Hand; SPADI, Shoulder Pain and Disability Index; FIM, Functional Independence Measure; ADLs, Activities of Daily Living; IQR, Interquartile Range; SD, Standard Deviation; CI, Confidence Interval.

Interaction effects calculated using two-way ANOVA (age × tear size).

## Discussion

This study aimed to examine the association between the timing of PT initiation and functional recovery following arthroscopic rotator cuff repair, with particular emphasis on PT adherence and key patient subgroups. The findings demonstrated that earlier initiation of PT was consistently associated with superior clinical outcomes across a broad spectrum of functional domains, including reduced disability, enhanced range of motion, lower pain levels, and greater functional independence. High adherence to prescribed PT regimens further amplified these benefits, showing a clear dose–response relationship between the extent of rehabilitation engagement and patient-reported as well as clinician-rated outcomes. Regression analysis confirmed that early PT and higher adherence were independent predictors of better functional recovery, even after adjusting for demographic and clinical covariates. However, it is important to acknowledge that the early PT group also demonstrated significantly higher adherence levels, which may partially mediate the relationship between initiation timing and outcome. Although regression modeling adjusted for adherence, residual confounding cannot be excluded, and the causal contribution of each factor remains interdependent. A linear mixed-effects model would offer a more robust framework for disentangling these effects in studies involving repeated or hierarchical data structures. Future investigations should incorporate such models to jointly account for timing, adherence, and patient-specific covariates within a single analytical framework. Subgroup analysis revealed that older age and larger tear size were linked to poorer outcomes, with a significant interaction effect indicating compounded impairment in older individuals with more extensive tears. Collectively, these results underscore the importance of early and sustained rehabilitation and highlight the influence of age and tear severity on postoperative trajectories.

The observed association between early initiation of physical therapy and improved functional outcomes following arthroscopic rotator cuff repair is likely attributable to the biological and neuromuscular advantages conferred by prompt rehabilitation (25). However, not all studies have demonstrated long-term advantages of early rehabilitation. For example, Cuff and Pupello (26) conducted a prospective randomized trial and found no significant difference in long-term outcomes between early and delayed PT groups, raising concerns about potential risks to tendon healing integrity. These discrepancies may reflect differences in surgical techniques, tear sizes, or rehabilitation protocols, and highlight the need to balance early mobilization with biological healing timelines (26). Further longitudinal studies are warranted to evaluate whether early gains in function persist without compromising structural outcomes over extended follow-up periods. Early mobilization facilitates tendon-to-bone healing by promoting collagen alignment, reducing postoperative stiffness, and preserving proprioception and neuromuscular control, whereas delayed therapy may contribute to fibrosis and joint contracture, impairing range of motion and muscular recovery (25, 27, 28). Additionally, patients who initiated therapy earlier completed more sessions and demonstrated greater adherence, reinforcing the cumulative therapeutic effect of consistent, supervised rehabilitation during the critical recovery window (6). These findings are consistent with prior studies. Kjær et al. (9) reported that early passive motion improved short-term function without compromising tendon integrity. McBroom et al. (29) also found that earlier physical therapy was associated with superior pain relief and mobility. Mazuquin et al. (7), in a systematic review, concluded that delayed rehabilitation offered no added benefit and was sometimes linked to increased stiffness. Burns et al. (30) emphasized that adherence to rehabilitation protocols significantly influences functional recovery, a relationship further supported by the present findings.

The clinical benefits associated with higher physical therapy adherence are underpinned by both physiological and behavioral mechanisms (31). Frequent therapy sessions promote neuromuscular re-education, joint mobilization, and progressive strengthening, which are critical for restoring shoulder function (28). Consistent engagement also enhances therapist-patient interaction and supports long-term adherence to home-based exercises (32). The dose–response relationship observed across adherence levels reinforces the importance of sustained rehabilitation intensity (30). These findings are supported by previous literature. Hall et al. (33) demonstrated that structured rehabilitation adherence improved both subjective and objective outcomes. Galetta et al. (6) and Stein et al. (34) similarly reported better recovery trajectories in patients who completed more of their prescribed therapy. Sakurai et al. (35) noted that therapist-rated compliance and patient-reported home exercise adherence predicted postoperative strength and range of motion (35). Collectively, these studies support the present findings and affirm the critical role of consistent rehabilitation in optimizing outcomes after rotator cuff repair.

### Clinical significance

The clinical significance of this study lies in its robust demonstration that both the timing and adherence to physical therapy critically influence postoperative recovery following arthroscopic rotator cuff repair. Specifically, initiating physical therapy within the early postoperative period and maintaining high adherence to prescribed rehabilitation protocols were independently associated with superior functional outcomes, including reduced disability, improved shoulder range of motion, enhanced functional independence, and shorter time to resume daily activities. These findings underscore the importance of integrating early and structured rehabilitation strategies into standard postoperative care pathways and highlight the need for clinicians to actively monitor and support patient adherence throughout the recovery process. Moreover, the identification of older age and larger tear size as modifiers of recovery trajectories reinforces the need for personalized rehabilitation planning in higher-risk subgroups.

### Limitations and areas of future research

This study has several limitations that warrant consideration. As a non-randomized observational design, it does not permit definitive causal inference regarding the effects of physical therapy timing and adherence on functional outcomes. Group allocation was based on the naturally occurring timing of rehabilitation initiation rather than randomization, introducing the potential for selection bias. Although baseline demographic and clinical characteristics were broadly comparable between groups, unmeasured confounders or factors facilitating earlier access to therapy may have influenced the observed associations. Adherence to supervised therapy and home exercise programs was partially self-reported, which may have introduced response bias. Functional outcomes were assessed at a single mid-term follow-up (3–6 months), limiting evaluation of long-term recovery trajectories and structural tendon integrity. Although preoperative MRI confirmed full-thickness rotator cuff tears, detailed morphological characteristics—such as fatty infiltration (Goutallier classification) and tendon retraction (Patte classification)—were not systematically graded or incorporated into the analyses; consequently, variability in baseline muscle–tendon unit quality, particularly among patients with large tears, could not be fully accounted for. The study population was restricted to individuals aged 40–70 years and was conducted at a single center, which may limit generalizability to other age groups or clinical settings. Future research should include randomized controlled trials with longitudinal follow-up, integrate standardized imaging-based structural classifications, and further examine sex-specific, socioeconomic, and psychological determinants of rehabilitation adherence to refine risk-adjusted and individualized postoperative care strategies.

## Conclusion

This study demonstrates that early initiation of physical therapy and higher adherence to postoperative rehabilitation protocols are independently associated with significantly better functional outcomes at 3–6 months following arthroscopic rotator cuff repair. These associations persisted after adjusting for age, tear size, comorbidities, and other clinical variables, with the largest effect observed for PT adherence. Subgroup analysis further revealed that older adults with larger tear sizes experience disproportionately worse outcomes, highlighting the compounded effect of age and injury severity on recovery. These findings support the clinical prioritization of early and sustained rehabilitation engagement as a modifiable determinant of improved postoperative function.

## Data Availability

The datasets presented in this study can be found in online repositories. The names of the repository/repositories and accession number(s) can be found in the article/supplementary material.

## References

[1] Harada Y Yokoya S Sumimoto Y Iwahori Y Kajita Y Deie M . Prevalence of rotator cuff tears among older tennis players and its impact on clinical findings and shoulder function. J Sport Rehabil. (2022) 31:849–55. doi: 10.1123/jsr.2021-0105, 35461187

[2] Li Y Yang S Cui L Bao Y Gu L Pan H . Prevalence, risk factor and outcome in middle-aged and elderly population affected by hemiplegic shoulder pain: an observational study. Front Neurol. (2023) 13:1041263. doi: 10.3389/fneur.2022.1041263, 36712437 PMC9879055

[3] Eckers F Loske S Ek ET Müller AM. Current understanding and new advances in the surgical management of reparable rotator cuff tears: a scoping review. J Clin Med. (2023) 12:1713. doi: 10.3390/jcm12051713, 36902499 PMC10003213

[4] Longo UG Carnevale A Piergentili I Berton A Candela V Schena E . Retear rates after rotator cuff surgery: a systematic review and meta-analysis. BMC Musculoskelet Disord. (2021) 22:749. doi: 10.1186/s12891-021-04634-6, 34465332 PMC8408924

[5] Brindisino F De Santis A Rossettini G Pellicciari L Filipponi M Rollo G . Post-surgery rehabilitation following rotator cuff repair. A survey of current (2020) Italian clinical practice. Disabil Rehabil. (2022) 44:4689–99. doi: 10.1080/09638288.2021.1916628, 33945358

[6] Galetta MD Keller RE Sabbag OD Linderman SE Fury MS Medina G . Rehabilitation variability after rotator cuff repair. J Shoulder Elb Surg. (2021) 30:e322–33. doi: 10.1016/j.jse.2020.11.016, 33418088

[7] Mazuquin B Moffatt M Gill P Selfe J Rees J Drew S . Effectiveness of early versus delayed rehabilitation following rotator cuff repair: systematic review and meta-analyses. PLoS One. (2021) 16:e0252137. doi: 10.1371/journal.pone.0252137, 34048450 PMC8162656

[8] Hu C-W Tsai SHL Chen C-H Tang H-C Su C-Y Tischler EH . Early versus delayed mobilization for arthroscopic rotator cuff repair (small to large sized tear): a meta-analysis of randomized controlled trials. BMC Musculoskelet Disord. (2023) 24:938. doi: 10.1186/s12891-023-07075-5, 38049792 PMC10694899

[9] Kjær BH Magnusson SP Henriksen M Warming S Boyle E Krogsgaard MR . Effects of 12 weeks of progressive early active exercise therapy after surgical rotator cuff repair: 12 weeks and 1-year results from the CUT-N-MOVE randomized controlled trial. Am J Sports Med. (2021) 49:321–31. doi: 10.1177/0363546520983823, 33471547

[10] Ziedas AC Castle JP Abed V Swantek AJ Rahman TM Chaides S . Race and socioeconomic status are associated with inferior patient-reported outcome measures following rotator cuff repair. Arthroscopy. (2023) 39:234–42. doi: 10.1016/j.arthro.2022.08.043, 36208711

[11] Fahy K Galvin R Lewis J Mc Creesh K. Exercise as effective as surgery in improving quality of life, disability, and pain for large to massive rotator cuff tears: a systematic review & meta-analysis. Musculoskelet Sci Pract. (2022) 61:102597. doi: 10.1016/j.msksp.2022.102597, 35724568

[12] Powell JK Costa N Schram B Hing W Lewis J. “Restoring that faith in my shoulder”: a qualitative investigation of how and why exercise therapy influenced the clinical outcomes of individuals with rotator cuff–related shoulder pain. Phys Ther. (2023) 103:pzad088. doi: 10.1093/ptj/pzad088, 37440455 PMC10733131

[13] Nayak MS Prabhu BA Balthillaya MG Ramachandra P Poojari DP Ts S . Exploring patient perspectives, socioeconomic status and beliefs on rehabilitation after arthroscopic rotator cuff repair: a qualitative study. Shoulder Elbow. (2025) 17:17585732251327175. doi: 10.1177/17585732251327175 PMC11948235 40160230

[14] Plancher KD Shanmugam J Briggs K Petterson SC. Diagnosis and management of partial thickness rotator cuff tears: a comprehensive review. J Am Acad Orthopaed Surg. (2021) 29:1031–43. doi: 10.5435/JAAOS-D-20-01092, 34520444

[15] Brindisino F Venturin D Bartoli M Caselli S Pellicciari L Poser A. Psychometric properties of the disability of arm shoulder and hand (DASH) in subjects with frozen shoulder: a reliability and validity study. BMC Musculoskelet Disord. (2024) 25:260. doi: 10.1186/s12891-024-07371-8, 38566086 PMC10986124

[16] Alotaibi NM Aljadi SH Alrowayeh HN. Reliability, validity and responsiveness of the Arabic version of the disability of arm, shoulder and hand (DASH-Arabic). Disabil Rehabil. (2016) 38:2469–78. doi: 10.3109/09638288.2015.1136846, 26856367

[17] Salvatore G Longo UG De Salvatore S Candela V Piergentili I Bandini B . Evaluating shoulder pain and disability index (SPADI) outcomes post-rotator cuff repair: minimal clinically important difference (MCID), patient acceptable symptom state (PASS) and substantial clinical benefit (SCB) analysis. J Back Musculoskelet Rehabil. (2025) 38:974–80. doi: 10.1177/10538127251320504 40138519

[18] Alsanawi HA Alghadir A Anwer S Roach KE Alawaji A. Cross-cultural adaptation and psychometric properties of an Arabic version of the shoulder pain and disability index. Int J Rehabil Res. (2015) 38:270–5. doi: 10.1097/MRR.0000000000000118, 25954858

[19] García-Rudolph A Wright M García L Sauri J Cegarra B Tormos JM . Long-term prediction of functional independence using adjusted and unadjusted single items of the functional independence measure (FIM) at discharge from rehabilitation. J Spinal Cord Med. (2024) 47:649–60. doi: 10.1080/10790268.2023.2183326, 36913541 PMC11378684

[20] Tashjian RZ Shin J Broschinsky K Yeh C-C Martin B Chalmers PN . Minimal clinically important differences in the American shoulder and elbow surgeons, simple shoulder test, and visual analog scale pain scores after arthroscopic rotator cuff repair. J Shoulder Elb Surg. (2020) 29:1406–11. doi: 10.1016/j.jse.2019.11.018, 32081634

[21] Ciardi G Novara D Quattrini F Ricci E. Rehabilitation outcome domains following rotator cuff surgical repair: a systematic review. J Orthop Rep. (2025) 4:100409. doi: 10.1016/j.jorep.2024.100409

[22] Scott W McCracken LM. Patients' impression of change following treatment for chronic pain: global, specific, a single dimension, or many? J Pain. (2015) 16:518–26. doi: 10.1016/j.jpain.2015.02.007, 25746196

[23] Christiansen DH Frost P Falla D Haahr JP Frich LH Svendsen SW. Responsiveness and minimal clinically important change: a comparison between 2 shoulder outcome measures. J Orthop Sports Phys Ther. (2015) 45:620–5. doi: 10.2519/jospt.2015.5760, 26110548

[24] Tang H Yang P Wang X Zhao B Ling K. Assessment of the efficacy of early versus delayed mobility exercise after arthroscopic rotator cuff repair. Int Orthop. (2025) 49:1411–20. doi: 10.1007/s00264-025-06477-5, 40053065 PMC12075342

[25] Chen Y Meng H Li Y Zong H Yu H Liu H . The effect of rehabilitation time on functional recovery after arthroscopic rotator cuff repair: a systematic review and meta-analysis. PeerJ. (2024) 12:e17395. doi: 10.7717/peerj.17395, 38784392 PMC11114118

[26] Cuff DJ Pupello DR. Prospective randomized study of arthroscopic rotator cuff repair using an early versus delayed postoperative physical therapy protocol. J Shoulder Elb Surg. (2012) 21:1450–5. doi: 10.1016/j.jse.2012.01.025, 22554876

[27] Lee C. Tendon physiology and repair. Orthop Trauma. (2021) 35:274–81. doi: 10.1016/j.mporth.2021.07.003

[28] Stephen J Flowers R Ho A Nakashima C Kasitinon D. "Postoperative rehabilitation". In: Musculoskeletal Pain: Evidence-Based Clinical Evaluation and Management Cham, Switzerland: Springer (2025). p. 513–96.

[29] McBroom TJ Abraham PF Varady NH Kucharik MP Eberlin CT Best MJ . Accelerated versus standard physical therapy in patients with transtendinous rotator cuff repair: a propensity-matched cohort study. J Shoulder Elb Surg. (2022) 31:S123–30. doi: 10.1016/j.jse.2021.10.039, 34864154

[30] Burns D Boyer P Razmjou H Richards R Whyne C. Adherence patterns and dose response of physiotherapy for rotator cuff pathology: longitudinal cohort study. JMIR Rehabil Assist Technol. (2021) 8:e21374. doi: 10.2196/21374, 33704076 PMC8082948

[31] Svingen J. Flexor Tendon Repair: Rehabilitation Adherence, Outcome and Complications. Stockholm: Karolinska Institutet (Sweden) (2022).

[32] Singh V Berry A Cramp F. Rotator cuff-related shoulder pain (RCRSP): semistructured patient interviews to explore the barriers and enablers to rehabilitation exercises. BMJ Open Sport Exerc Med. (2024) 10:e001978. doi: 10.1136/bmjsem-2024-001978, 39415878 PMC11481144

[33] Hall K Grinstead A Lewis JS Mercer C Moore A Ridehalgh C. Rotator cuff related shoulder pain. Describing home exercise adherence and the use of behavior change interventions to promote home exercise adherence: a systematic review of randomized controlled trials. Phys Ther Rev. (2021) 26:299–322. doi: 10.1080/10833196.2021.1935106

[34] Stein AM Hardy A Moussa M Bauer T Werthel JD. Higher pain catastrophizing scale is associated with more postoperative pain within the first week after rotator cuff repair. J Exp Orthopaed. (2025) 12:e70359. doi: 10.1002/jeo2.70359, 40655253 PMC12255946

[35] Sakurai T Yamazaki H Tomii K Takahashi Y Abe Y Kobayashi Y. Combined home and clinic-based therapy versus home-based exercise programme after distal radial fracture: a randomized controlled study. J Hand Surg Eur Vol. (2024) 49:1085–94. doi: 10.1177/17531934241231709, 38366383

